# The Isaac Knapp Dental Coterie

**Published:** 1892-05

**Authors:** 


### The Isaac Knapp Dental Coterie.

A few days ago we had the opportunity of learning, by observation, what a live city dental society is for the profession in its midst and in the vicinity.

It was my privilege to be present for one evening at a meeting of the *Isaac Knapp Dental Coterie,* of Ft. Wayne, Ind. This Society was organized some four or five years ago in memory of Dr. Isaac Knapp, the pioneer of true dental practice in that city; one who did more for the elevation of Dental Science and art in Northeastern Indiana than any other man up to the time of his death, and he left an influence and impress that will never pass away; and may this Society ever regard it as one of its most sacred duties to cherish and perpetuate his memory. His professional life was an inspiration to all who knew him and to all who may know of him, so far as they can appreciate his worth.

This Society is doing much to uphold and sustain the honor and character of the profession, as is shown by the appreciation entertained by the public of that city and vicinity for honorable dental service and those who render it.

The Society have it in contemplation and are considering the subject of establishing a Dental Infirmary, not for the purpose of teaching but purely as a matter of benevolence, for those whose circumstances will not permit them to have the service of the dentist in the regular way. This, when established, will prove a boon to the poor, and a great relief to the busy and overworked dentist. We will be pleased to refer to this matter more in detail when it is in full operation. Ft. Wayne is a model western city, it has a copious supply of the purest water we have ever seen in any city; it has an ample supply of natural gas, and the atmosphere is as clear and pure as crystal, and with these and the accompaniments who could not be happy.

Long may the *Isaac Knapp Dental Coterie* live, prosper, and stand as a model that others may imitate.

